# Machine learning to construct sphingolipid metabolism genes signature to characterize the immune landscape and prognosis of patients with uveal melanoma

**DOI:** 10.3389/fendo.2022.1056310

**Published:** 2022-12-08

**Authors:** Hao Chi, Gaoge Peng, Jinyan Yang, Jinhao Zhang, Guobin Song, Xixi Xie, Dorothee Franziska Strohmer, Guichuan Lai, Songyun Zhao, Rui Wang, Fang Yang, Gang Tian

**Affiliations:** ^1^ Clinical Medical College, Southwest Medical University, Luzhou, China; ^2^ School of Stomatology, Southwest Medical University, Luzhou, China; ^3^ Department of General, Visceral, and Transplant Surgery, Ludwig-Maximilians-University Munich, Munich, Germany; ^4^ Department of Epidemiology and Health Statistics, School of Public Health, Chongqing Medical University, Chongqing, China; ^5^ Department of Neurosurgery, Wuxi People’s Hospital Affiliated to Nanjing Medical University, Wuxi, China; ^6^ Department of Ophthalmology, Charité – Universitätsmedizin Berlin, Campus Virchow-Klinikum, Berlin, Germany; ^7^ Department of Laboratory Medicine, The Affiliated Hospital of Southwest Medical University, Luzhou, China

**Keywords:** sphingolipid metabolism, UVM, tumor microenvironment, immunotherapy, predictive signature

## Abstract

**Background:**

Uveal melanoma (UVM) is the most common primary intraocular malignancy in adults and is highly metastatic, resulting in a poor patient prognosis. Sphingolipid metabolism plays an important role in tumor development, diagnosis, and prognosis. This study aimed to establish a reliable signature based on sphingolipid metabolism genes (SMGs), thus providing a new perspective for assessing immunotherapy response and prognosis in patients with UVM.

**Methods:**

In this study, SMGs were used to classify UVM from the TCGA-UVM and GEO cohorts. Genes significantly associated with prognosis in UVM patients were screened using univariate cox regression analysis. The most significantly characterized genes were obtained by machine learning, and 4-SMGs prognosis signature was constructed by stepwise multifactorial cox. External validation was performed in the GSE84976 cohort. The level of immune infiltration of 4-SMGs in high- and low-risk patients was analyzed by platforms such as CIBERSORT. The prediction of 4-SMGs on immunotherapy and immune checkpoint blockade (ICB) response in UVM patients was assessed by ImmuCellAI and TIP portals.

**Results:**

4-SMGs were considered to be strongly associated with the prognosis of UVM and were good predictors of UVM prognosis. Multivariate analysis found that the model was an independent predictor of UVM, with patients in the low-risk group having higher overall survival than those in the high-risk group. The nomogram constructed from clinical characteristics and risk scores had good prognostic power. The high-risk group showed better results when receiving immunotherapy.

**Conclusions:**

4-SMGs signature and nomogram showed excellent predictive performance and provided a new perspective for assessing pre-immune efficacy, which will facilitate future precision immuno-oncology studies.

## Introduction

UVM accounts for 85% of all ocular melanomas and is the most common primary intraocular malignancy in adults (1). Approximately 85% of tumor cases arise from the choroid, with the remaining cases arising from the iris (3-5%) and ciliary body (5-8%) (2, 3). More than 50% of patients with UVM develop systemic metastatic disease, with the liver being the most common site of metastasis in UVM (4). In addition, patients rarely achieve a good cure with surgery (5). As a result, patients with UVM have a very poor prognosis, with a 5-year mortality rate of 31% and a 15-year mortality rate of 45% (6). ICBhe diameter of the basal tumor, ciliary involvement and scleral expansion, non-random chromosomal aberrations and genetic mutations (e.g., BAP1 and SF3B1 mutations) are closely related to the prognosis of UVM (7–9) and are the main basis and foundation for prognostic grading, immunotherapy, radiotherapy and other treatment options. However, patients with the same clinical stage may have different clinicopathological features, suggesting that the prognosis of tumor patients based on traditional clinicopathological staging is not completely accurate (10, 11). Therefore, to improve the quality of life of UVM patients, new prognostic biomarkers and molecular targets are needed to predict the prognosis of UVM patients and guide individualized treatment.

Sphingolipids are important components of biological membrane structure, maintaining the barrier function and fluidity of cell membranes (12). With the intensive study of sphingolipids in animals and yeast, sphingolipids and their metabolites have been found to be an important class of bioactive molecules, which are involved in regulating many important signaling processes such as cell growth, differentiation, senescence and programmed cell death (13). There is increasing evidence that sphingolipid metabolism is extensively involved in tumor proliferation, metastasis, angiogenesis and drug resistance, and plays a key role in the tumor immune microenvironment (14–18). In addition, it has been suggested that sphingolipids may be a potential tumor-associated antigen and are closely associated with tumor evolution and liver metastasis in UVM (19). Pelletier et al. found that UVM contains sphingolipids, which may be a target for monoclonal antibody therapy (20). With the continuous development of bioinformatics, biomarkers have been defined in various ways. Due to the unique role of sphingolipid metabolism in the tumor microenvironment, several studies have identified the potential of sphingolipid-related genes or SMGs in the prognosis prediction of tumor tumors with a high degree of accuracy (21–24). Currently, the prognostic value of SMGs in UVM and the role of tumor immune microenvironment are unclear. Therefore, this study aimed to develop a novel SMGs-based approach to accurately predict prognosis and characterize the immune landscape of UVM patients.

In our study, we screened 4 reliable SMGs by machine learning, constructed a prognostic model based on the TCGA-UVM cohort, and went on to establish a risk score and comprehensively analyze the relationship between SMGs and immune microenvironment, immunotherapy, and chemotherapy sensitivity. We aimed to demonstrate the value of 4-SMGs for assessing the prognosis of UVM patients through a comprehensive analysis of genomic data, and to develop new tools to improve treatment options.

## Method

### Patient data sources

We downloaded gene expression profiles and clinical data of TCGA-UVM cohort including 80 tumor patients from TCGA database (https://portal.gdc.cancer.gov/). The level 3 HTSeq-Fragments per kilobase million (FPKM) data of TCGA-UVM was converted to TPM (transcripts per million reads) according to the following formula: TPMn = FPKMn * 10^6^/(FPKM0 +… + FPKMm), where n represented gene n and m represented the total number of all genes, respectively. Then, we performed log_2_-based transformation of TPM. The sample size of UVM patients at the M stage and N stage varied greatly. These stages were consequently excluded from the analysis. The gene profiles and clinical data of 28 UVM patients in GSE84976 dataset were downloaded from the GEO database (https://www.ncbi.nlm.nih.gov/geo/). The GSE84976 was considered as an external validation dataset.

### Consensus clustering analysis

To further elucidate the SMGs signature in UVM, all samples were divided into different clusters using “ConensusClusterPlus” R package (25). The “pheatmap” R package was used to show the differential expression and clinicopathological parameters of SMGs in different clusters. Gene set variation analysis (GSVA) analysis was performed using “c2.cp.kegg.v7.5.1.symbols.gmt” from the MSigDB database. Analysis of pathway differences across clusters using the “GSVA” R package (26). The single sample gene set enrichment analysis (ssGSEA) algorithm (27) was used to analyze the level of immune cell infiltration and the level of immune checkpoint expression between different clusters.

### Model construction and validation

We obtained 97 SMGs (**Supplementary Table 1**) through the InnateDB portal (http://www.innatedb.com) (28). By performing univariate Cox regression analysis, we identified 27 genes associated with survival, followed by Least absolute shrinkage and selection operator (LASSO) regression analysis using ‘glmnet’ in R, with tenfold cross-validation to determine the optimal penalty parameter lambda.min=9. Nine genes were obtained. Support vector machine recursive feature elimination (SVM-RFE) is another machine learning method that uses the structural risk minimization principle while minimizing the empirical error as a way to improve learning performance (29). We used the SVM-RFE algorithm from the ‘e1071’ R package, with ten-fold cross-validation to obtain 13 valuable variables. Five public genes were extracted by Wayne diagram analysis, followed by using a stepwise multifactor COX regression model to identify and calculate the coefficients of the core genes. Finally, the risk signature of 4-SMGs was constructed. For each patient, the SMGs risk score was calculated as follows, risk score = Expression_mRNA1_ × Coef_mRNA1_ + Expression_mRNA2_ × Coef_mRNA2_ +… Expression_mRNAn_ × Coef_mRNAn_.

### Model formula

All UVM patients were given risk scores based on output model equations, and median value were calculated using the R package “survminer”, classifying all UVM patients into low-risk and high-risk groups, and plotting survival curves for the two subgroups. The R package “pec” was adopted to calculate the C-index. For assessing genetic traits’ predictive power, receiver operating characteristic curve (ROC) curve analysis using the “time-ROC” R package was conducted. Decision curve analysis (DCA) of a multi-factor Cox regression model was plotted using the “ggDCA” R package.

### Independent prognostic analysis and nomogram construction

We conducted univariate and multivariate Cox regression analyses to assess risk score as an independent prognostic factor. Using the “rms” R package, histograms were constructed using risk scores versus clinicopathologic characteristics to predict survival for patients in TCGA-UVM cohort.

### Functional enrichment analysis

Through functional enrichment analysis of differentially expressed genes in UVM associated with SMGs, functional annotation and enrichment pathways have been explored. The analysis of Gene Ontology (GO) pathways was done using the “ClusterProfiler” R package, where P-value < 0.05 represents a statistically significant difference. GSVA was performed using “c2.cp.kegg.v7.5.1.symbols.gmt” from the MSigDB. Using “GSVA” R package to perform GSVA enrichment analysis. The “heatmap” R package was used to create heat maps. According to the “limma” R package, an adjusted P-value < 0.05 indicates statistical significance for subgroup differences.

### Immunity analysis of the risk signature

Currently accepted methods, including XCELL (30, 31), TIMER (32, 33), QUANTISEQ (32, 33), MCPCOUNT (34), EPIC (35), CIBERSORT (36) and CIBERSORT-ABS (37) were used to measure immune infiltration scores. Spearman correlation analysis was used to examine the correlation between immune cells and risk scores. Based on the immune cell characteristics of UVM patients, the ssGSEA method was adopted to differentiate patients at low-risk from those at high-risk. Using a list of 20 suppressive immune checkpoints derived from Auslander’s study, we assessed the suppression of immune checkpoints between high-risk and low-risk groups (38). The “estimate” R package was used to calculate the immunological and mechanistic scores of the specimens from the RNA-seq data to assess the purity of the tumors. Evaluation and visualization of immunotherapy efficacy in UVM patients by “limma” and “ggpubr” R package.

Xu et al. developed a website that provided us with gene sets related to cancer and immunity (39) (http://biocc.hrbmu.edu.cn/TIP/) and a set of genes positively associated with anti-PD-L1 drug response was obtained from Mariathasan’s study features (40). The R package “ggcor” for the analysis of correlations between risk scores and the two genetic traits mentioned above was used. ImmuCellAI (http://bioinfo.life.hust.edu.cn/ImmuCellAI) (41) is a portal that predicts tumor immune infiltration estimates and immunotherapy response, and we obtained corresponding data on immunotherapy in UVM patients.

### Drug sensitivity

The “pRRophetic” R package was used to assess treatment response in high-risk and low-risk groups of patients, as determined by the half-maximal inhibitory concentration (IC50) of each UVM patient on the Genomics of Drug Sensitivity in Cancer (GDSC) (https://www.cancerrxgene.org/) (42).

### TISCH analysis

A single-cell RNA sequencing database focused on TME is housed at the Tumor Immunization Single Cell Center (TISCH). Detailed cell type annotations are provided at the single cell level for further analysis of specific gene expression in different cell types. The specific gene expression in different cell types further reveals the variation of TME in patients with different UVM, thus explaining to some extent the heterogeneity of UVM.

### Statistical analysis

Statistical analyses were performed using R software v4.1.3. Kaplan-Meier (KM) survival curves and log-rank test were used to compare Overall Survival (OS) between high- and low-risk groups. LASSO regression analysis and SVM-RFE for screening candidate SMGs. Stepwise multi-factor cox regression analysis was used to construct SMGs signature. Time-dependent ROC was used to evaluate the predictive performance of the model. Spearman correlation analysis was used to evaluate the correlation between risk score and immune cell infiltration. Wilcox test was used to compare the proportion of TIICs, immune checkpoints, and immune function between the two groups. P-values <0.05 were considered statistically significant and false discovery rate (FDR)<0.05 was considered statistically significant.

## Result

### Consensus clustering identified the molecular subtypes of SMGs

**Figure 1:**
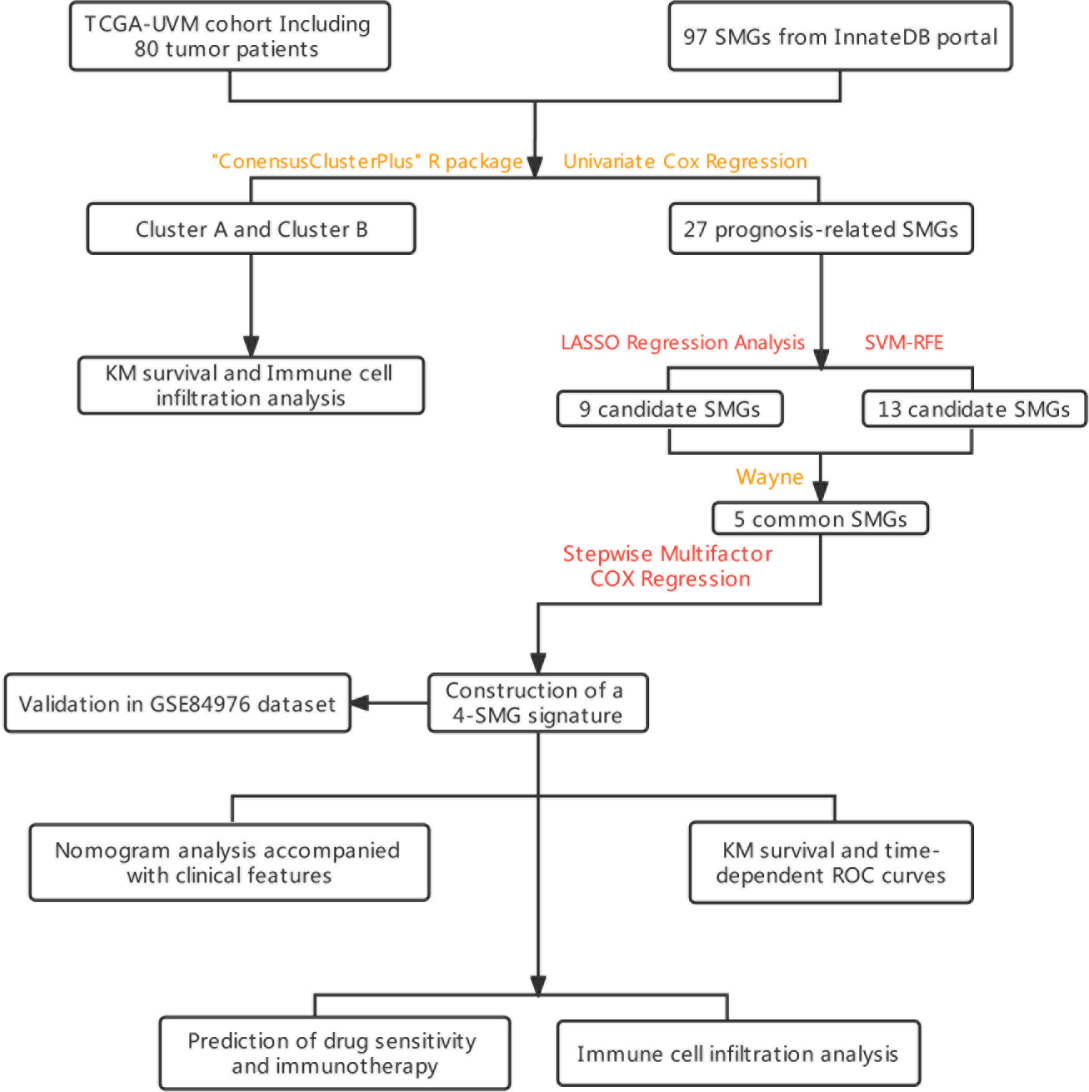
The flowchart summarizes the main design of the present study.

**Figure 2:**
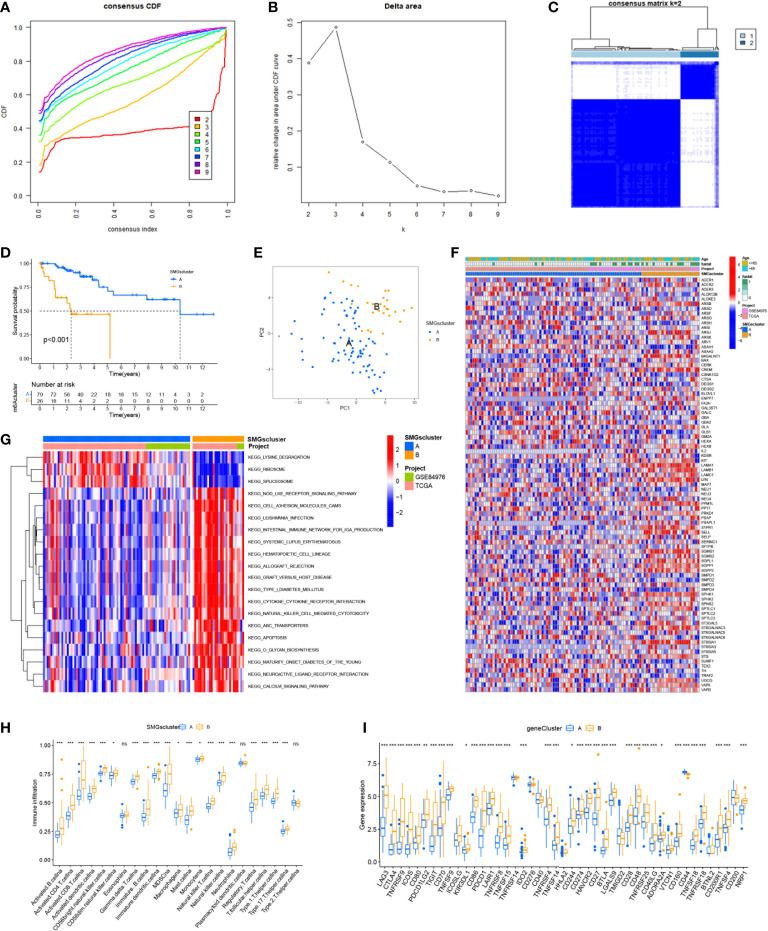
Consensus clustering identified the molecular subtypes of SMGs. **(A)** Consensus clustering CDF for k = 2 to 9. **(B)** Relative change in area under the cumulative CDF curve for k = 2 to 9. **(C)** Consensus matrix for k = 2. **(D)** The different OS between Cluster A and Cluster B. **(E)** PCA plot . **(F)** Relationships between SMGs expression and clinicopathological parameters. **(G)** KEGG enrichment analysis of different clusters. * *P* < 0.05, ** *P* < 0.01, *** *P* < 0.001, ns>0.05. **(H)** Immune cell scores between Cluster A and Cluster B. **(I)** Different expressions of immune checkpoints between Cluster A and Cluster B.

The graphical flow chart outlines the main design of this study (**Figure 1**). We considered that the increasing trend of the cumulative distribution function (CDF) values relative to the consensus index indicated the presence of appropriate classification, and based on the CDF curve and the Delta area, k = 2 proved to be the best point to obtain the maximum difference between clusters when the clustering index “k” increased from 2 to 9, so we divided the UVM patients into two subgroups (**Figures 2A, B**). In addition, the consensus matrix is naturally a better visualization tool that can help to assess the composition and number of clusters. We plotted the color-coded heat map corresponding to the consensus matrix and found that it exhibited high intra-group correlation and low inter-group correlation when k=2, which strongly suggests that it is very appropriate to classify UVM patients into two subtypes (Cluster A and Cluster B) (**Figure 2C**).

To determine the difference in survival prognosis of patients in different clusters, the difference in OS between clusters was calculated according to the ClusterSurvival R package. We found an improved survival prognosis in cluster A compared to patients in cluster B (*P*<0.001) (**Figure 2D**). Principal component analysis (PCA) is often used to visualize the distribution of risk in different populations. Cluster A and Cluster B patients showed significant differences when based on different clusters (**Figure 2E**). In addition to this, we further explored the metabolic differences between Cluster A, B and SMGs, and the heat map showed that Cluster B had higher expression differences and clinical characteristics in SMGs (**Figure 2F**). To elucidate potential biological pathways, we performed enrichment analysis of different cluster samples using the Kyoto Encyclopedia of Genes and Genomes (KEGG) pathway database and identified relationships with various cancer-related pathways, such as apoptosis, transporters, cell adhesion molecules and hematopoietic cell lineages (**Figure 2G**). Based on the fact that immunotherapy plays an important role in the treatment of tumors, to understand the distribution and correlation of the relative content of 23 TIICs (tumor-infiltrating immune cells) in this cohort, we calculated the level of immune cell infiltration in both clusters by the ssGSEA algorithm. It was found that there was a higher level of infiltration in most immune cells in Cluster B compared to Cluster A (**Figure 2H**). According to the results, the SMGs risk score model can classify different immune subtypes and thus influence the response to immunotherapy. Furthermore, due to the importance of immune checkpoints for the effectiveness of tumor immunotherapy and the fact that immune checkpoints are one of the important features of tumor microenvironment (TME). We explored the differences in immune checkpoint expression between the two groups and ultimately found that immune checkpoint gene expression was significantly upregulated in patients with Cluster B. Based on the above analysis, we concluded that Cluster B has higher effectiveness and sensitivity for immunotherapy (**Figure 2I**).

### Construction and validation of 4-SMGs signature.

**Figure 3:**
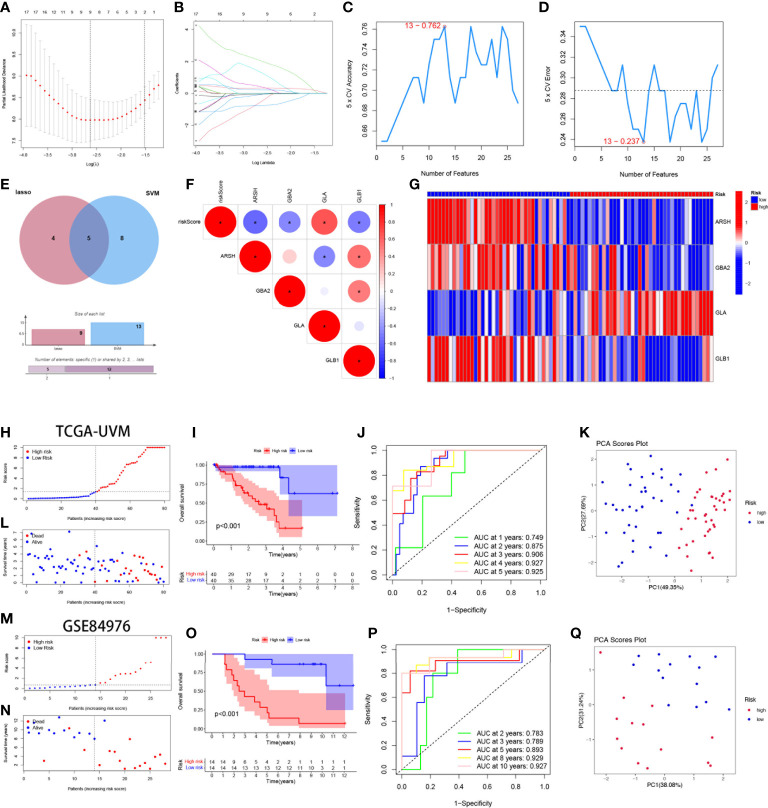
Construction and validation of 4-SMGs Signature. **(A)** Ten‐time cross‐validation for tuning parameter selection in the LASSO model. **(B)** LASSO coefficient profiles. **(C, D)** Biomarker signature gene expression validation by SVM-RFE algorithm selection. **(E)** Two algorithmic venn diagram screening genes. **(F)** Correlation of 4-SMGs with riskscore. **(G)** Heatmap of risk factors in high- and low-risk patients. **(H)** Distribution of risk scores between low- and high-risk groups in the TCGA cohort. **(I)** Survival status of UVM patients in the low- and high-risk groups in the TCGA cohort. **(J)** KM curve compares the overall UVM patients between low- and high-risk groups in the TCGA cohort. **(K)** Time-dependent ROC curves analysis in the TCGA cohort. **(L)** PCA plot in the TCGA cohort. **(M)** Distribution of risk scores between low- and high-risk groups in the GEO cohort. **(N)** Survival status of UVM patients in the low- and high-risk groups in the GEO cohort. **(O)** KM curve compares the overall UVM patients between low- and high-risk groups in the GEO cohort. **(P)** Time-dependent ROC curves analysis in the GEO cohort. **(Q)** PCA plot in the GEO cohort.

By univariate cox regression analysis, we obtained a total of 27 SMGs significantly associated with OS in UVM patients (**Supplementary Table 2**). We used two machine learning methods to identify candidate SMGs. For the LASSO regression analysis, 9 candidate genes were screened from the 27 SMGs with significant prognostic features (**Figures 3A, B**). For the SVM-RFE algorithm, the error was minimized when the number of features was 13 (**Figures 3C, D**). Five intersecting genes were obtained by Wayne diagrams for the above two methods (**Figure 3E**). Finally, 4 SMGs were identified as independent prognostic factors by the stepwise multivariate Cox analysis, including ARSH, GBA2, GLA and GLB1. Prognostic index (PI) = (-0.701*ARSH exp) + (-3.988*GBA2 exp) + (5.464*GLA exp) + (-2.985*GLB1 exp). We further explored the correlation between the expression of these 4-SMGs and risk scores and found that all 4-SMGs were closely associated with risk scores. Among them, ARSH, GBA2 and GLB1 had a significant negative correlation with the risk score, while GLA had a significant positive correlation with the risk score (**Figure 3F**). In addition, we calculated the prognostic risk score for each patient and divided the UVM patients into high-risk and low-risk groups based on the median score of score. (**Figure 3G**) demonstrates the distribution of 4-SMGs in the high- and low-risk groups, with ARSH, GBA2 and GLB1 being low expressed in patients in the high-risk group and GLA being highly expressed in high-risk patients. In the TGCA-UVM cohort, mortality in UVM patients increased with increasing risk (**Figures 3H, I**), with a better prognosis in the low-risk group (*P*<0.001) (**Figure 3J**). Time-dependent ROC curves were used to assess the accuracy of the model developed to predict OS in UVM patients. The time-dependent ROC curves showed 1-year AUC of 0.749, 2-year AUC of 0.875, 3-year AUC of 0.906, 4-year AUC of 0.927, and 5-year AUC of 0.925 (**Figure 3K**). PCA is often used to visualize the distribution of risk in different populations. When based on a risk model, high- and low-risk patients showed significant differences and showed a clear separation (**Figure 3L**). In the GSE84976 cohort, we demonstrated the same results as in the TCGA-UVM cohort. mortality in UVM patients increased with increasing risk (**Figures 3M, N**). KM survival analysis showed that low-risk patients had a better prognosis compared to high-risk patients (*P*<0.001) (**Figure 3O**). The time-dependent ROC curve showed a 2-year AUC of 0.783, a 3-year AUC of 0.789, a 5-year AUC of 0.893, an 8-year AUC of 0.929and a 10-year AUC of 0.927 (**Figure 3P**). PCA analysis showed that low- and high-risk patients exhibited significant differences, showing a clear separation (**Figure 3Q**). Based on these results, we can conclude that the construction of our prognostic model is quite superior.

### Clinical correlation and survival analysis of SMGs in UVM patients

**Figure 4:**
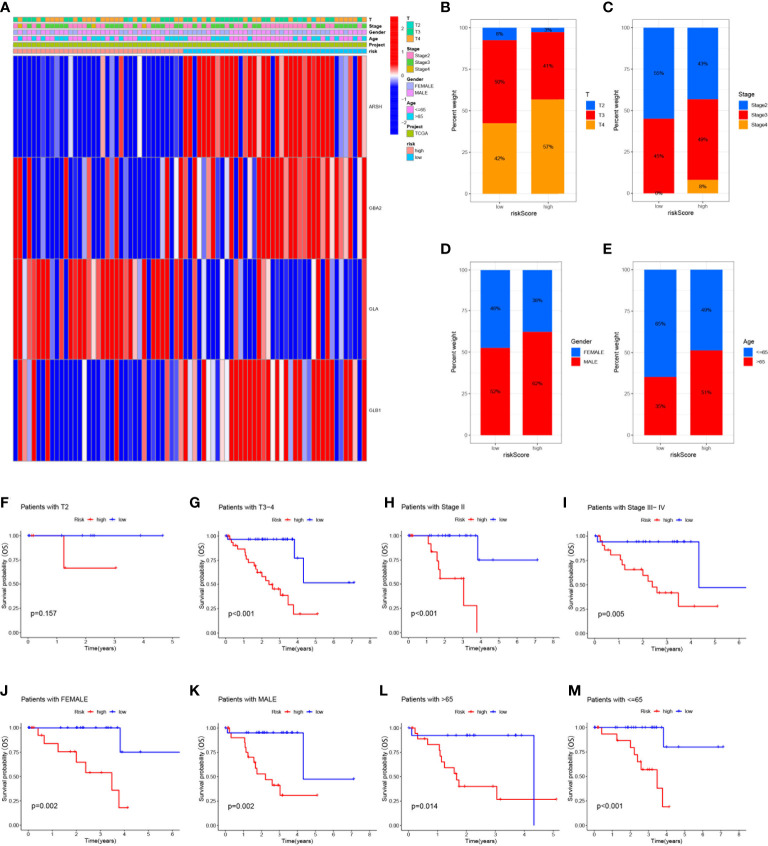
Clinical correlation and survival analysis of SMGs in patients with UVM **(A)** heatmap showing the associations between the high-risk and low-risk score of patients with UVM and UVM related clinical characteristics in the dataset from TCGA. **(B-E)** The percentage Stacked histogram of clinical characteristics. The risk score based on 4-SMGs signature is a valuable marker for poor prognosis in various subgroups divided by clinicopathological characteristics. The SMGs could distinguish high-risk patients in a variety of subgroups divided by clinicopathological characteristics including **(F, G)** T stage, **(H, I)** clinical stage, **(J, K)** gender and **(L, M)** age.

To analyze the correlation between high- and low-risk groups and clinical characteristics, heat maps were drawn based on the expression of clinical characteristics, risk scores, and 4-SMGs, the heat maps showed the association between the 4-SMGs identified in the prognostic risk model and the age, gender, clinical stage, T stage, and risk scores of all UVM patient samples in TCGA (**Figure 4A**). In addition, we further analyzed the difference in the proportion of patients with various clinicopathological characteristics between the high-risk and low-risk groups and found that SMGs had a significant impact on the proportion of patients with different clinicopathological characteristics (**Figures 4B–E**). To better understand whether the prognosis of patients in different clinical subgroups differed, a clinical analysis was performed on the entire sample subgroup, all samples were divided into different subgroups by age (>65 and ≤65 years), gender (male and female), clinical stage (II and III-IV) and T stage (T2 and T3-4) for further survival analysis. Survival times were significantly shorter in high-risk patients than in low-risk patients in all subgroups (**Figures 4F–M**). The currently identified risk model for SMGs also seems to be able to reliably predict the prognosis of certain subgroups of UVM based on their clinical characteristics.

### Establishment of nomograms in combination with clinical characteristics

**Figure 5:**
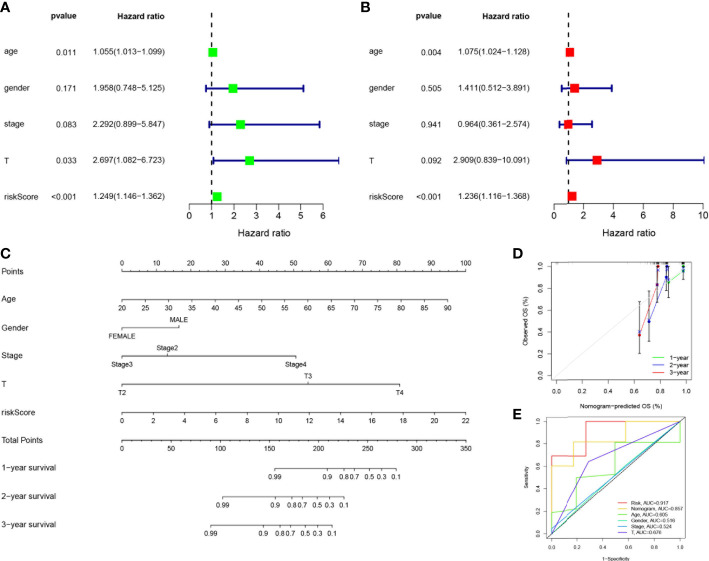
Establishment of nomograms in combination with clinical characteristics. **(A)** Univariate and **(B)** multivariate COX regression analysis of the signature and different clinical feature. Green squares: univariate cox analysis of HR values for each variable. Red squares: multivariate cox analysis of HR values for each variable. **(C)** A nomogram combining the SMGs, age, gender, clinical stage, and T stage. **(D**) The calibration curve of the constructed nomogram of 1-year, 2-year and 3-year survival. **(E)** The nomogram’s time-dependent ROC curves.

Considering the strong correlation between the constructed risk model and poor prognosis, we combined the OS of UVM patients and their clinical characteristics in univariate and multivariate Cox analyses to determine whether our prognostic characteristics constructed based on the 4-SMGs could be used as independent predictors of prognosis. Based on the results of univariate analysis of variables, age, stage, gender, T, and risk score, risk score was found to be significantly associated with prognosis in patients with UVM (*P*<0.001) (**Figure 5A**). Similarly, risk score remained the most reliable and independent predictor in the cohort after multifactorial analysis *(P*<0.001) (**Figure 5B**). To extend the clinical application and usability of the constructed risk model, we constructed Nomogram plots based on age, gender, clinical stage, T stage, and risk score as a predictor of 1-, 2-, and 3-year prognostic survival probabilities in patients with UVM. As a result, it was observed from the model results that the risk score had the greatest impact on predicting OS, indicating that the SMGs-based risk model could better predict the prognosis of UVM (**Figure 5C**). The calibration curves also showed a more satisfactory consensus between the predicted and observed values in terms of the probability of OS at 1-, 2- and 3- years (**Figure 5D**). Comparing Nomogram, risk and common clinicopathological features again, risk (AUC=0.917), as well as Nomogram (AUC=0.857), were better predictors of UVM prognosis than age, gender, clinical stage and T stage(**Figure 5E**). This implies that the predictive performance of our constructed SMGs signature was significantly better than other clinical features.

### Functional enrichment analysis of SMGs

**Figure 6:**
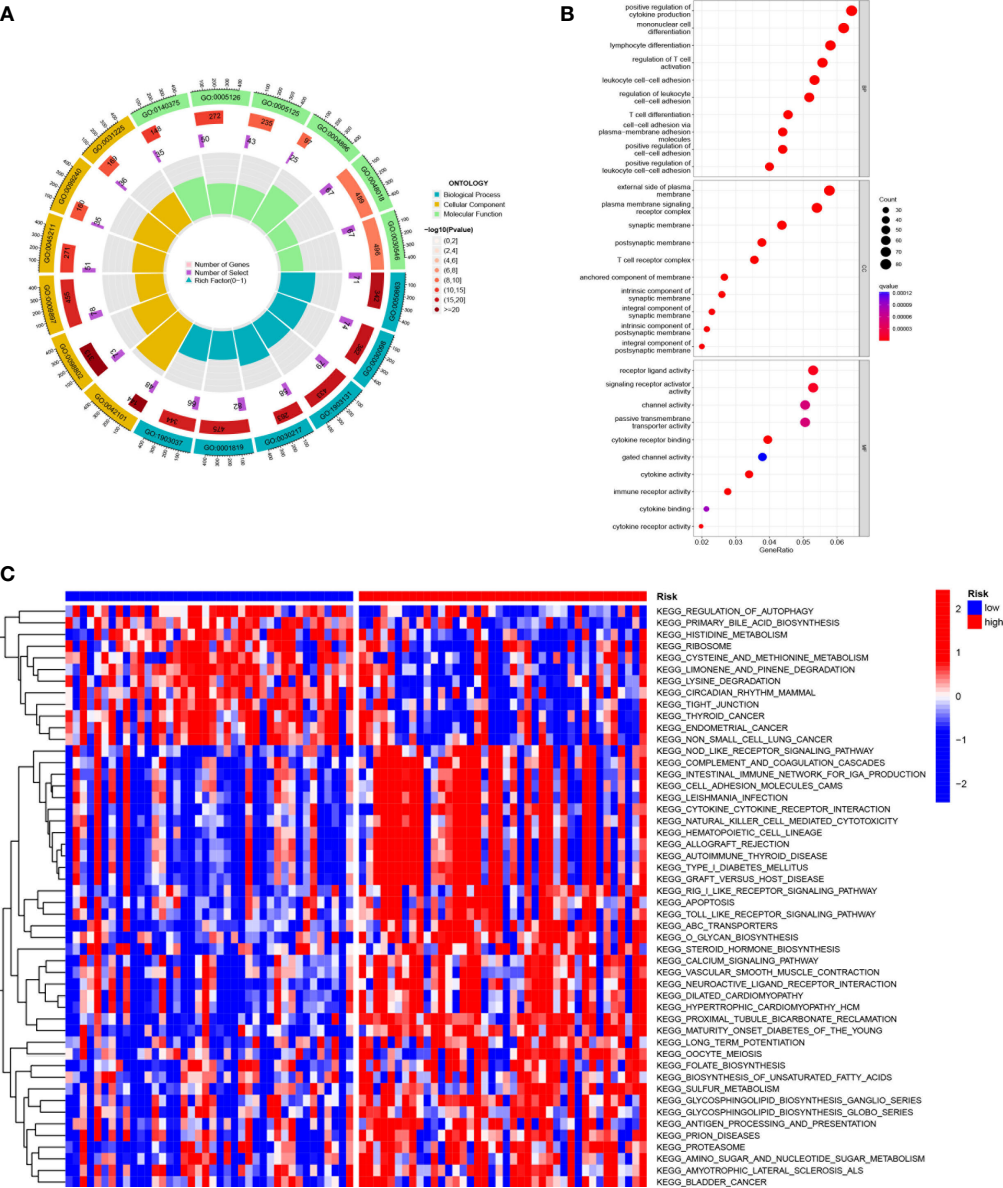
Functional enrichment analysis of SMGs in TCGA-UVM. **(A, B)** Gene Ontology (GO) enrichment analysis was used to analyze the differential genes between UVM and normal samples. The x axis represents the ratio of genes associated with the term, and the y axis represents the pathway term. The p value of each item is colored according to the legend. **(C)** GSVA analysis between the high-risk cohort and the low-risk cohort with KEGG.

KEGG enrichment analysis and GO functional analysis were performed to assess differential genes in UVM to elucidate the relevance of bioactivity and signaling pathways to risk scores. The threshold FDR<0.05 and *P*<0.05 were used to select significantly enriched items (**Figures 6A, B**; **Supplementary Table 3**). Biological processes (BP) mainly included such as lymphocyte and T-cell differentiation, leukocyte adhesion, etc. The cellular component (CC) mainly included the plasma membrane signal transduction receptor complex, synaptic membrane intrinsic components and plasma membrane signal transduction receptor complex. Molecular function (MF) mainly included receptor ligand activity, immune receptor activity, cytokine binding, and signaling receptor activator activity. GSVA analysis identified 186 significantly enriched pathways (**Figure 6C**; **Supplementary Table 4**), and among low-risk individuals, cystine and methionine metabolism, regulation of autophagy, and cancer-related pathways were enriched, while in the high-risk group, pathway enrichment mostly involved immune function, including natural killer cell-mediated cytotoxicity, T-cytokine cytokine receptor interaction, sterol hormone biosynthesis and other related KEGG pathways. In summary, we were surprised to find a strong correlation between enrichment analysis results and immune response, and therefore we conducted a systematic analysis of the immune landscape in the UVM patients.

### SMGs risk score predicts TME and immune cell infiltration

**Figure 7:**
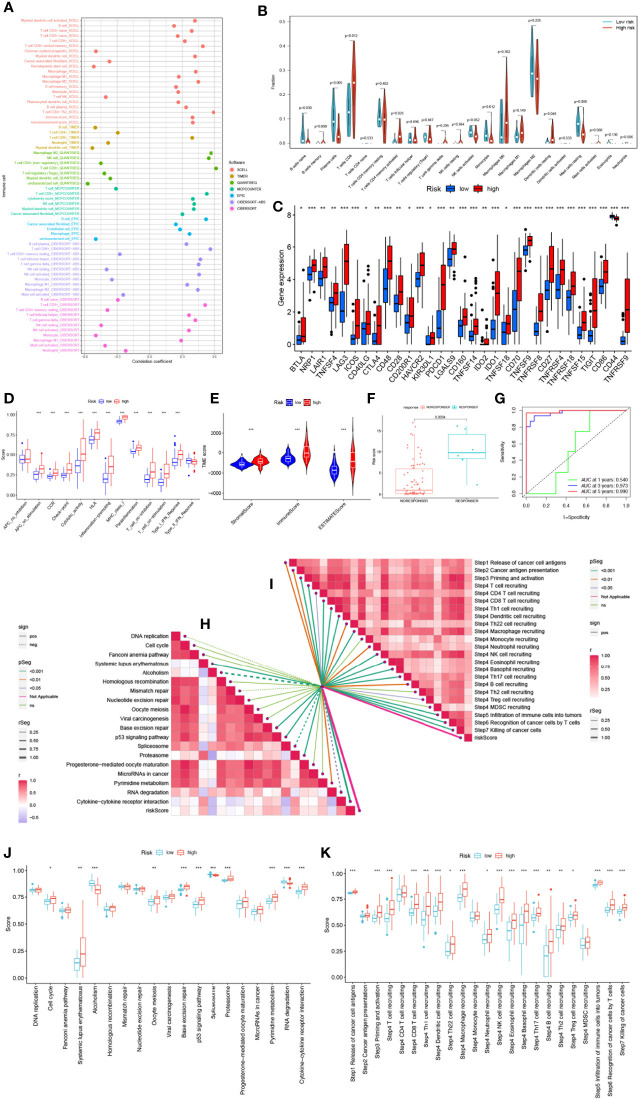
SMGs risk score predicts TME and immune cell infiltration. **(A)** Immune cell bubble map. **(B)** Differences in immune cell infiltration between high- and low-risk groups. **(C)** Immune checkpoint differences between high and low risk groups. **(D)** Immune cell and immune function ssGSEA scores between high- and low-risk groups. **(E)** The TME component analysis. **(F)** 4-SMGs predict immunotherapy response outcomes in patients. **(G)** Excellent predictive performance of ROC curve labeling model **(H)** Correlation between risk score and ICB response signature. **(I)** Correlation of risk scores with each step of the tumor immune cycle. **(J)** The plot of the difference in enrichment scores between the high-risk and low-risk groups on the immunotherapy prediction pathway. **(K)** The plot of differences between the high-risk and low-risk groups on each step of the cancer-immune cycle. * *P <*0.05; ** *P <*0.01; *** *P <*0.001.

Crosstalk between cancer cells and the TME has been proven to play an important role in tumor progression and metastasis (43). And TIICs are an important component of the TME, and their composition and distribution are closely related to tumorigenesis and development (44). First, we explored the correlation between risk score and infiltrating immune cell abundance according to XCELL, TIMER, QUANTISEQ, MCPCOUNTER, CIBERSORT, CIBERSORT-ABS and EPIC algorithms, where the CD8+ T cell, NK cell infiltration were all positively correlated with the risk score (**Figure 7A** **).** We then independently assessed immune cell infiltration between high and low-risk groups in UVM using the CIBERSORT algorithm and showed that the expression of plasma cells, B cells naïve, Monocytes, and Mast cells resting was significantly higher in the high-risk group (**Figure 7B**). Given the importance of checkpoint-based immunotherapy, we analyzed the expression of immune checkpoint genes in the high- and low-risk groups. Most of the immune checkpoint genes were found to be significantly upregulated in the high-risk group, including IDO1, CTLA-4, TIGIT, KIR3DL1, BTLA, CD28, etc. (**Figure 7C**), suggesting that patients in the high-risk group may have better efficacy with ICB therapy. Because immune cells with immune checkpoints can significantly affect immune function, we performed a comparison of ssGSEA scores for immune function, and multiple immune function scores were significantly greater in the high-risk group than in the low-risk group (**Figure 7D**). As infiltrating immune cells are an important component and one of the characteristics of the TME, alterations in the expression of immune cell types lead to changes in TME composition, so we analyzed the TME composition of UVM samples. The results showed that the immune score (*P*<0.001), ESTIMATE score (*P*<0.001) and Stromal Score (*P*<0.001) were lower in the low-risk group compared with the high-risk group, indicating that the overall immune level and immunogenicity of the TME were higher in the high-risk group (**Figure 7E**). More importantly, we obtained immunotherapy response outcomes for UVM patients through the immuneAI portal and found that patients with higher risk scores were more likely to benefit from immunotherapy (**Figure 7F**), and had a better predictive performance for 4-SMGs signature (**Figure 7G**). Since ICB response plays an important role in immune checkpoint therapy, we further analyzed the correlation between risk score and ICB response signature (**Figures 7H, J** **)** and found that among them, Systemic lupus erythematosus, Base excision repair, p53 signaling pathway, Proteasome, and Cytokine-cytokine receptor interaction were significantly positively correlated, while significantly negatively correlated with Alcoholism and Spliceosome. The correlation analysis between risk score and tumor immune cycle steps was also performed, and it was found that only MDSC recruiting, Th2 cell recruiting, and Monocyte recruiting (step 4) were not significantly correlated with risk score, while other immune cycle steps were positively correlated with our risk score (**Figures 7I, K**).

### 4-SMGs signature predicts chemotherapy sensitivity

**Figure 8:**
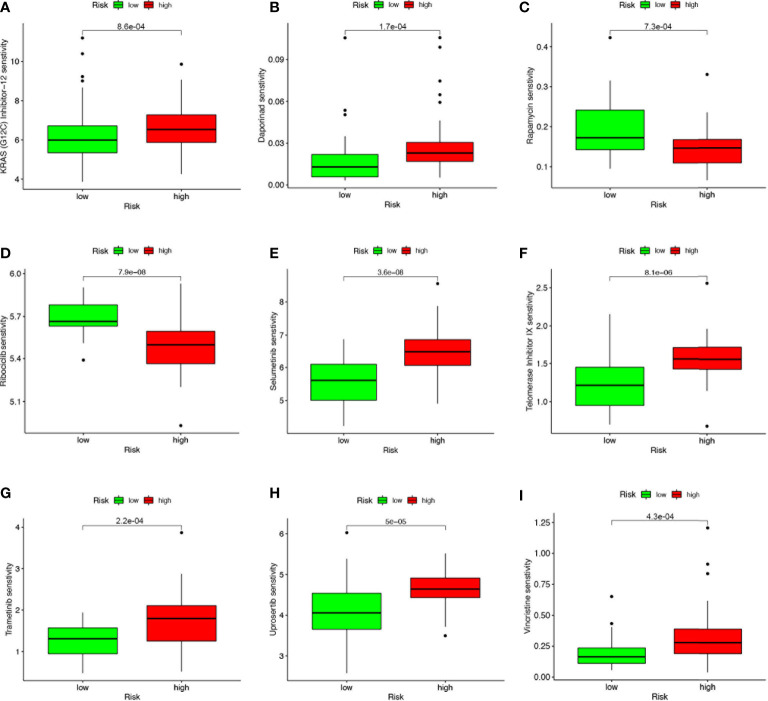
4-SMGs signature predicts chemotherapy sensitivity. **(A)** KRAS (G12C) Inhibitor-12, **(B)** Daporinad, **(C)** Rapamycin, **(D)** Rapamycin, **(E)** Selumetinib, **(F)** Telomerase Inhibitor IX, **(G)** Trametinib, **(H)** Uprosertib and **(I)** Vincristine.

Based on the risk score, we further evaluated the potential value of 4-SMGs in predicting chemotherapy sensitivity through the GDSC database to enhance accurate drug therapy. A total of 51 chemotherapeutic or targeted agents with significant differences in chemosensitivity between high and low risk were analyzed by the “pRRophetic” R package (**Supplementary Figure 1**). **Figure 8** showed nine common inhibitors or drugs. KRAS (G12C) Inhibitor-12, Daporinad, Selumetinib, Telomerase Inhibitor IX, Trametinib, Uprosertib and Vincristine had relatively high IC50 in the high-risk group. In contrast, Rapamycin and Rapamycin had lower IC50s in the high-risk group.

### Correlation of SMGs with tumor microenvironment

**Figure 9:**
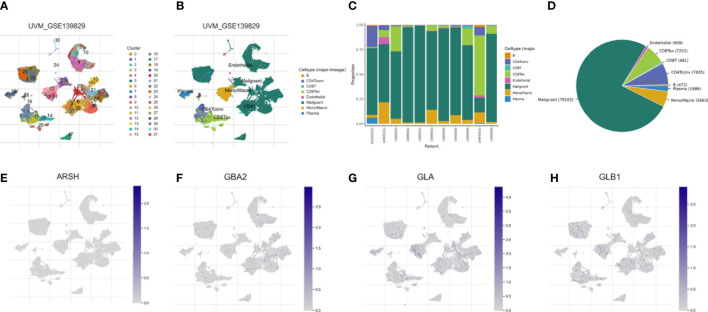
Correlation of SMGs with tumor microenvironment. **(A–D)** Annotation of all cell types in UVM_GSE139829 and the percentage of each cell type. Percentages and expressions of **(E)** ARSH, **(F)** GBA2, **(G)** GLA and **(H)** GLB1.

We used the single-cell dataset UVM_GSE139829 from the TISCH database to analyze the expression of 4-SMGs in the immune microenvironment. There were 31 cell populations and 8 immune cell types in the UVM_GSE139829 dataset (**Figures 9A, B**), showing the distribution and number of various cell types (**Figures 9C, D**). Expression levels of each SMGs in immune cells were barely expressed in ARSH in the immune microenvironment (**Figure 9E**), GBA2, GLA, and GLB1 were expressed in various immune cells (**Figures 9F–H**). GLA was mainly expressed in Mono/Marco.

## Discussion

Despite its low incidence, UVM is still the most common primary intraocular malignant tumor and is known for its high metastasis, high malignancy, and high mortality (45). Surgical removal is the first-line treatment of UVM. Although it has a great impact on the appearance and psychology of patients, the prognosis of UVM patients after surgery is also unsatisfactory, and the five-year survival rate is only 17%-53% (46). In addition, the 5-year and 10-year metastasis rates of UVM are about 25% and 34%, respectively. The mortality rate of UVM within 1 year after metastasis is 80%. Most patients with metastatic UVM have a survival time of 6-12 months after diagnosis, and there are almost no relevant management strategies and treatment measures for metastatic UVM (47–49). Therefore, the early diagnosis and treatment of UVM are particularly important to improve the prognosis of patients. The diagnosis and prognosis prediction of UVM is based on clinical manifestations and histopathological evaluation, but they are not enough to judge the heterogeneity and development trend of tumors. At the same time, they have limitations in predicting the prognosis and treatment response of UVM patients, while molecular prognosis prediction methods have better performance (50). Sphingolipid metabolism is a highly regulated intracellular process that controls the synthesis and degradation of bioactive lipids, including ceramide and sphingosine-1-phosphate (51). A large number of studies have explored the relationship between sphingolipids and cancer. We noted that gangliosides in sphingolipids were closely associated with liver metastasis and host immune response in a human UVM nude mouse model and that anti-gangliosides antibodies could inhibit the spread of tumor cells in nude mice (52, 53). In addition, sphingolipid metabolites promote tumor progression by promoting cell proliferation and stimulating chemotactic migration and invasion, such as SP1 in ovarian cancer (54–57). This is exciting and may imply that sphingolipids play an important role in UVM proliferation, metastasis, and phenotype and can be used as a target for monoclonal antibodies and immunotherapy. Unfortunately, there are still gaps in this research. Given the great potential of sphingolipids for UVM, we used mRNA expression data from the TCGA-UVM dataset to identify important prognostic genes and constructed a multi-biomarker prognostic model based on SMGs. Our results suggest that SMGs-based signatures can be used for risk stratification, prognosis prediction and immunotherapy efficacy assessment of UVM, thus providing a valuable reference for individualized treatment.

In this study, we integrated the expression profiles of 97 SMGs in the TCGA-UVM dataset and selected four genes (ARSH, GBA2, GLA and GLB1) to construct a new prognostic model for SMGs by using Lasso regression analysis, SVM-RFE, and stepwise multivariate COX regression analysis. The SMGs signature we constructed proved to be an independent prognostic factor for UVM. UVM patients were divided into high and low risk groups according to the median risk score, and significant prognostic differences were found between the two groups. In other reports, non-lysosomal β-glucosidase (GBA2) hydrolyzed GlcCer to release ceramide and glucose on the cytoplasmic side of ER and golgi membrane, potentially affecting tumor drug resistance (58, 59). Alpha-galactosidase (GLA) has long been considered a key link in Fabry’s disease (60), but has rarely been studied in tumors, which seems to provide a way to link Fabry’s disease and tumors. Lysosomal β-galactosidase (GLB1) hydrolyzed β-galactosidase from a sugar conjugate, representing the origin of the aging-associated β-galactosidase SA-SS-GAL, was reported to be a reliable biomarker of aging in prostate cancer (61). However, other studies have shown that the expression of lysosomal galactosidase β-1 (GLB1) is not associated with aging and is active in some types of cancer cells (62). The effect of GLB1 on various types of tumors is still inconclusive, and a large number of related mechanism studies are needed to elucidate. ROC curve and calibration curve analysis proved the outstanding predictive performance of SMGs signature. In order to expand the predictive ability of SMGs signature and prove its practical value in assessing the prognosis of UVM patients, we constructed a nomogram based on clinical factors and risk scores and found that SMGs signature has better predictive efficacy than clinicopathological features, which can provide a basis for clinicians to make decisions.

In order to have a comprehensive and clear demonstration of SMGs signature function, we carried out enrichment analysis of action pathways. The results showed that SMGs signature was associated with many immune or tumor related pathways, such as monocyte differentiation, lymphocyte differentiation, T cell differentiation, regulation of T cell activation, cell adhesion, cytokine and its receptor activity, etc. The cytotoxicity of lymphocytes to UVM cells with hematologic metastasis may imply that SMGs can promote lymphocyte differentiation to prevent UVM from migrating along the blood. Lentz et al. found that monocyte and melanocyte destruction invaded the uveal tract in UVM patients (63), suggesting that SMGs are associated with primary changes in UVM. UVM cells express PD-L1 to regulate T cell function by inhibiting IL-2 production, thus achieving immune escape (64). SMGs may activate T cell function, thereby achieving better efficacy of PD-1/PD-L1 targeted therapy. The enrichment of various adhesion pathways suggests that SMGs play an important role in UVM metastasis. We found many interesting results when we performed KEGG enrichment analysis. The low-risk group is enriched in the autophagy regulatory pathway, but the specific mechanism of this aspect is lacking. Given the importance of autophagy for tumor behavior and treatment, this represents a huge research prospect. UVM is protected from complement mediated cleavage *in vitro* and *in vivo* by expressing three types of CRP (CD46, CD55, and CD59) (65), while the high-risk group is enriched in complement related pathways. Therefore, it is hypothesized that patients in the high-risk group may prevent the immune escape of UVM by stimulating complement, but experimental verification is needed, which will be our next research direction.

The TME plays a key role in the occurrence, progression, metastasis and treatment resistance of tumors (66). Immune components in the TME play a key role in promoting immune escape and inflammation formation (67). A deeper understanding of immune infiltration in TME is essential to reveal the underlying molecular mechanisms and provide novel immunotherapy strategies to improve clinical outcomes (68–71). Hence, We analyzed the immune cell infiltration in the high - and low-risk groups. Activated naive B cells have been shown to promote Th1 polarization, thereby preventing further tumor growth (72). Plasma cells are the end functional state of the B-cell lineage and are involved in solid tumor progression. A large number of literature have shown that tumor-infiltrating plasma cells have a positive prognostic effect on cancer (73, 74). The increased expression of naive B cells and plasma cells in the low-risk group may indicate a better prognosis in the low-risk group. In addition, we also noted that mast cell infiltration was significantly higher in the low-risk group than in the high-risk group. Mast cells have many effects on tumors, including mast cell-mediated cytotoxicity, mast cell-directed angiogenesis, tissue remodeling in the adjacent environment, and immune cell recruitment (75). Some studies have suggested that mast cells have a great influence on tumor angiogenesis in UVM (76). However, the actual role of mast cells in UVM and the specific mechanism still need a lot of experiments to explain. The high-risk group has higher infiltration of CD8+T cells. It has been found that the major histocompatibility complex II (MHC II) UVM vaccine can activate tumor-specific CD8+T cells and CD4+T cells, thereby killing tumor cells but does not affect normal cells, suggesting that the high-risk group receiving the MHC II UVM vaccine may have better efficacy (77). DC cocultured with UVM cells showed reduced expression of CD1a and CD83, which failed to activate T cells for immune response, revealing the possibility of tumor-pulsed dendritic cell vaccines as therapeutic measures for UVM (78). In addition, the degree of infiltration of resting dendritic cells in the high-risk group was higher than that in low-risk group, but there was no significant difference in activated dendritic cells, which may imply that UVM in high-risk group can change the status of dendritic cells to achieve immune escape.

With the increasing attention to tumor immune checkpoint, immunotherapy based on immune checkpoint inhibitors also has a good performance in clinical practice. But for UVM, immune checkpoint therapy has little effect (79). Some studies have pointed out that the retrospective data show that the response rate is very low (80). Therefore, there is an urgent need to analyze the expression of immune checkpoint genes in patients to identify patients who can benefit from immune checkpoint therapy. We found that the expression of most immune checkpoints was higher in the high-risk group compared with the low-risk group, suggesting that the high-risk group is more likely to benefit from ICB therapy. However, due to the rarity of UVM, the sample size limits the applicability and generality of almost all relevant studies, and there is still a need to increase the sample and conduct randomized prospective trials (81). In general, SMGs model can accurately identify UVM patients who are sensitive to ICB therapy, which has guiding significance for clinicians’ immunotherapy strategy. Given the influence of immune cells and immune checkpoints on immune function, we further explored the differences in immune function between high and low-risk groups. We found that HLA expression was higher in the high-risk group than in the low-risk group. It is generally assumed that low HLA expression will allow tumor cells to escape from CTL-mediated lysis, resulting in metastasis. However, a large number of studies have shown that the role of HLA in UVM is contrary to that of other common tumors. UVM cells reduce the expression of HLA antigens during liver metastasis through the blood route, thereby escaping the killing effect mediated by NK cells and cytotoxic T cells (82–84). This provides a new direction for the treatment of high-risk patients by increasing the expression of HLA antigens in UVM cells. Interestingly, we also found that the type II interferon response was also enhanced in the high-risk group. Bosch et al. developed an MHC II -matched vaccine that induces type II interferon secretion, thereby allowing the generation of a CD8 cell immune response in the eye (85) and may be more effective in high-risk patients.

Although our study has greater clinical implications for the prognostic assessment and selection of treatment options for patients with UVM, our study still has some limitations. First, our study is a retrospective study that needs to be validated in future prospective studies. Second, the mechanisms by which SMGs affect the prognosis of UVM patients need to be explored in more *in vivo* and *in vitro* experiments. Finally, the predominant race in the TCGA-UVM cohort was white, with a lack of data on Asians or blacks. This makes it critical to include other ethnic groups in future studies.

## Data availability statement

The original contributions presented in the study are publicly available. This data can be found here: https://github.com/zsylittlecloud/R-code-for-1056310-Machine-learning-to-construct-sphingolipid-metabolism-genes-signature-to-chara.git.

## Author contributions

HC, FY and GT conceived the study. HC, GP, JY, JZ, XX and GS drafted the manuscript. HC, DS and RW performed the literature search and collected the data. HC, GL, XX and SZ analyzed and visualized the data. FY and GT helped with the final revision of this manuscript. All authors reviewed and approved the final manuscript.

## References

[1] Chang AE Karnell LH Menck HR . The national cancer data base report on cutaneous and noncutaneous melanoma: A summary of 84,836 cases from the past decade. the American college of surgeons commission on cancer and the American cancer society. Cancer (1998) 83:1664–78. doi: 10.1002/(SICI)1097-0142(19981015)83:8<1664::AID-CNCR23>3.0.CO;2-G 9781962

[2] McLaughlin CC Wu XC Jemal A Martin HJ Roche LM Chen VW . Incidence of noncutaneous melanomas in the U. S. Cancer (2005) 103:1000–7. doi: 10.1002/cncr.20866 15651058

[3] Damato B . Progress in the management of patients with uveal melanoma. the 2012 Ashton lecture. Eye (Lond) (2012) 26:1157–72. doi: 10.1038/eye.2012.126 PMC3443832 22744385

[4] Lorigan JG Wallace S Mavligit GM . The prevalence and location of metastases from ocular melanoma: imaging study in 110 patients. AJR Am J Roentgenol (1991) 157:1279–81. doi: 10.2214/ajr.157.6.1950883 1950883

[5] Javed A Milhem M . Role of natural killer cells in uveal melanoma. Cancers (Basel) (2020) 12:3694. doi: 10.3390/cancers12123694 33317028 PMC7764114

[6] Kujala E Mäkitie T Kivelä T . Very long-term prognosis of patients with malignant uveal melanoma. Invest Ophthalmol Vis Sci (2003) 44:4651–9. doi: 10.1167/iovs.03-0538 14578381

[7] Yue H Qian J Yuan Y Zhang R Bi Y Meng F . Clinicopathological characteristics and prognosis for survival after enucleation of uveal melanoma in Chinese patients: Long-term follow-up. Curr Eye Res (2017) 42:759–65. doi: 10.1080/02713683.2016.1245422 27911584

[8] Dogrusöz M Jager MJ . Genetic prognostication in uveal melanoma. Acta Ophthalmol (2018) 96:331–47. doi: 10.1111/aos.13580 29105334

[9] Griewank KG Koelsche C van de Nes JAP Schrimpf D Gessi M Möller I . Integrated genomic classification of melanocytic tumors of the central nervous system using mutation analysis, copy number alterations, and DNA methylation profiling. Clin Cancer Res (2018) 24:4494–504. doi: 10.1158/1078-0432.CCR-18-0763 29891723

[10] Chi H Jiang P Xu K Zhao Y Song B Peng G . A novel anoikis-related gene signature predicts prognosis in patients with head and neck squamous cell carcinoma and reveals immune infiltration. Front Genet (2022) 13:984273. doi: 10.3389/fgene.2022.984273 36092898 PMC9459093

[11] Chi H Peng G Wang R Yang F Xie X Zhang J . Cuprotosis programmed-Cell-Death-Related lncRNA signature predicts prognosis and immune landscape in PAAD patients. Cells (2022) 11:3436. doi: 10.3390/cells11213436 36359832 PMC9658590

[12] Hannun YA Obeid LM . Principles of bioactive lipid signalling: lessons from sphingolipids. Nat Rev Mol Cell Biol (2008) 9:139–50. doi: 10.1038/nrm2329 18216770

[13] Spiegel S Kolesnick R . Sphingosine 1-phosphate as a therapeutic agent. Leukemia (2002) 16:1596–602. doi: 10.1038/sj.leu.2402611 12200669

[14] Ogretmen B . Sphingolipid metabolism in cancer signalling and therapy. Nat Rev Cancer (2018) 18:33–50. doi: 10.1038/nrc.2017.96 29147025 PMC5818153

[15] Modrak DE . Measurement of ceramide and sphingolipid metabolism in tumors: potential modulation of chemotherapy. Methods Mol Med (2005) 111:183–94. doi: 10.1385/1-59259-889-7:183 15911980

[16] Kreitzburg KM van Waardenburg R Yoon KJ . Sphingolipid metabolism and drug resistance in ovarian cancer. Cancer Drug Resist (2018) 1:181–97. doi: 10.20517/cdr.2018.06 PMC6936734 31891125

[17] Hawkins CC Ali T Ramanadham S Hjelmeland AB . Sphingolipid metabolism in glioblastoma and metastatic brain tumors: A review of sphingomyelinases and sphingosine-1-Phosphate. Biomolecules (2020) 10:1357. doi: 10.3390/biom10101357 32977496 PMC7598277

[18] Janneh AH Ogretmen B . Targeting sphingolipid metabolism as a therapeutic strategy in cancer treatment. Cancers (Basel) (2022) 14:2183. doi: 10.3390/cancers14092183 35565311 PMC9104917

[19] Tardif M Coulombe J Soulières D Rousseau AP Pelletier G . Gangliosides in human uveal melanoma metastatic process. Int J Cancer (1996) 68:97–101. doi: 10.1002/(SICI)1097-0215(19960927)68:1<97::AID-IJC17>3.0.CO;2-3 8895547

[20] Soulieres D Rousseau A Deschenes J Tremblay M Tardif M Pelletier G . Characterization of gangliosides in human uveal melanoma cells. Int J Cancer (1991) 49:498–503. doi: 10.1002/ijc.2910490404 1917148

[21] da Silva G de Matos LL Kowalski LP Kulcsar M Leopoldino AM . Profile of sphingolipid-related genes and its association with prognosis highlights sphingolipid metabolism in oral cancer. Cancer Biomark (2021) 32:49–63. doi: 10.3233/CBM-203100 34092610 PMC12500039

[22] Kim SJ Lee JH Park WJ Kim S . Bioinformatic exploration for prognostic significance of sphingolipid metabolism-related genes in invasive ductal carcinoma using the cancer genome atlas cohort. Int J Gen Med (2021) 14:4423–34. doi: 10.2147/IJGM.S328376 PMC8370849 34413672

[23] Hu X Zhou X Zhang J Li L . Sphingolipid metabolism is associated with osteosarcoma metastasis and prognosis: Evidence from interaction analysis. Front Endocrinol (Lausanne) (2022) 13:983606. doi: 10.3389/fendo.2022.983606 36105405 PMC9465041

[24] Sun Y Xu Y Che X Wu G . Development of a novel sphingolipid signaling pathway-related risk assessment model to predict prognosis in kidney renal clear cell carcinoma. Front Cell Dev Biol (2022) 10:881490. doi: 10.3389/fcell.2022.881490 35846357 PMC9277577

[25] Wilkerson MD Hayes DN . ConsensusClusterPlus: a class discovery tool with confidence assessments and item tracking. Bioinformatics (2010) 26:1572–3. doi: 10.1093/bioinformatics/btq170 PMC2881355 20427518

[26] Hänzelmann S Castelo R Guinney J . GSVA: Gene set variation analysis for microarray and RNA-seq data. BMC Bioinf (2013) 14:7. doi: 10.1186/1471-2105-14-7 PMC3618321 23323831

[27] Barbie DA Tamayo P Boehm JS Kim SY Moody SE Dunn IF . Systematic RNA interference reveals that oncogenic KRAS-driven cancers require TBK1. Nature (2009) 462:108–12. doi: 10.1038/nature08460 PMC2783335 19847166

[28] Breuer K Foroushani AK Laird MR Chen C Sribnaia A Lo R . InnateDB: systems biology of innate immunity and beyond–recent updates and continuing curation. Nucleic Acids Res (2013) 41:D1228–33. doi: 10.1093/nar/gks1147 PMC3531080 23180781

[29] Sanz H Valim C Vegas E Oller JM Reverter F . SVM-RFE: selection and visualization of the most relevant features through non-linear kernels. BMC Bioinf (2018) 19:432. doi: 10.1186/s12859-018-2451-4 PMC6245920 30453885

[30] Aran D Hu Z Butte AJ . xCell: Digitally portraying the tissue cellular heterogeneity landscape. Genome Biol (2017) 18:220. doi: 10.1186/s13059-017-1349-1 29141660 PMC5688663

[31] Aran D . Cell-type enrichment analysis of bulk transcriptomes using xCell. Methods Mol Biol (2020) 2120:263–76. doi: 10.1007/978-1-0716-0327-7_19 32124326

[32] Chen B Khodadoust MS Liu CL Newman AM Alizadeh AA . Profiling tumor infiltrating immune cells with CIBERSORT. Methods Mol Biol (2018) 1711:243–59. doi: 10.1007/978-1-4939-7493-1_12 PMC5895181 29344893

[33] Li T Fu J Zeng Z Cohen D Li J Chen Q . TIMER2.0 for analysis of tumor-infiltrating immune cells. Nucleic Acids Res (2020) 48:W509–14. doi: 10.1093/nar/gkaa407 32442275 PMC7319575

[34] Dienstmann R Villacampa G Sveen A Mason MJ Niedzwiecki D Nesbakken A . Relative contribution of clinicopathological variables, genomic markers, transcriptomic subtyping and microenvironment features for outcome prediction in stage II/III colorectal cancer. Ann Oncol (2019) 30:1622–9. doi: 10.1093/annonc/mdz287 PMC6857614 31504112

[35] Racle J de Jonge K Baumgaertner P Speiser DE Gfeller D . Simultaneous enumeration of cancer and immune cell types from bulk tumor gene expression data. Elife (2017) 6:e26476. doi: 10.7554/eLife.26476 29130882 PMC5718706

[36] Zhang H Li R Cao Y Gu Y Lin C Liu X . Poor clinical outcomes and immunoevasive contexture in intratumoral IL-10-Producing macrophages enriched gastric cancer patients. Ann Surg (2022) 275:e626–35. doi: 10.1097/SLA.0000000000004037 32541216

[37] Tamminga M Hiltermann TJN Schuuring E Timens W Fehrmann RS Groen HJ . Immune microenvironment composition in non-small cell lung cancer and its association with survival. Clin Transl Immunol (2020) 9:e1142. doi: 10.1002/cti2.1142 PMC7291326 32547744

[38] Auslander N Zhang G Lee JS Frederick DT Miao B Moll T . Robust prediction of response to immune checkpoint blockade therapy in metastatic melanoma. Nat Med (2018) 24:1545–9. doi: 10.1038/s41591-018-0157-9 PMC6693632 30127394

[39] Xu L Deng C Pang B Zhang X Liu W Liao G . TIP: A web server for resolving tumor immunophenotype profiling. Cancer Res (2018) 78:6575–80. doi: 10.1158/0008-5472.CAN-18-0689 30154154

[40] Mariathasan S Turley SJ Nickles D Castiglioni A Yuen K Wang Y . Powles, TGFβ attenuates tumour response to PD-L1 blockade by contributing to exclusion of T cells. Nature (2018) 554:544–8. doi: 10.1038/nature25501 PMC6028240 29443960

[41] Miao YR Zhang Q Lei Q Luo M Xie GY Wang H . ImmuCellAI: A unique method for comprehensive T-cell subsets abundance prediction and its application in cancer immunotherapy. Adv Sci (Weinh) (2020) 7:1902880. doi: 10.1002/advs.201902880 32274301 PMC7141005

[42] Geeleher P Cox NJ Huang RS . Clinical drug response can be predicted using baseline gene expression levels and *in vitro* drug sensitivity in cell lines. Genome Biol (2014) 15:R47. doi: 10.1186/gb-2014-15-3-r47 24580837 PMC4054092

[43] Xia Z Qing B Wang W Gu L Chen H Yuan Y . Formation, contents, functions of exosomes and their potential in lung cancer diagnostics and therapeutics. Thorac Cancer (2021) 12:3088–100. doi: 10.1111/1759-7714.14217 PMC8636224 34734680

[44] Tan Z Fu S Feng R Huang Y Li N Wang H . Identification of potential biomarkers for progression and prognosis of bladder cancer by comprehensive bioinformatics analysis. J Oncol (2022) 2022:1802706. doi: 10.1155/2022/1802706 35498536 PMC9042640

[45] Mei S Li Y Kang X . Prognostic and functional analysis of NPY6R in uveal melanoma using bioinformatics. Dis Markers (2022) 2022:4143447. doi: 10.1155/2022/4143447 35432628 PMC9012612

[46] Sheng X Li S Chi Z Si L Cui C Mao L . Prognostic factors for conjunctival melanoma: a study in ethnic Chinese patients. Br J Ophthalmol (2015) 99:990–6. doi: 10.1136/bjophthalmol-2014-305730 25595173

[47] Krantz BA Dave N Komatsubara KM Marr BP Carvajal RD . Uveal melanoma: epidemiology, etiology, and treatment of primary disease. Clin Ophthalmol (2017) 11:279–89. doi: 10.2147/OPTH.S89591 PMC5298817 28203054

[48] Straatsma BR Diener-West M Caldwell R Engstrom RE . Mortality after deferral of treatment or no treatment for choroidal melanoma. Indian J Ophthalmol (2018) 66:1395–400. doi: 10.4103/ijo.IJO_1499_18 PMC6173014 30249822

[49] Triozzi PL Singh AD . Adjuvant therapy of uveal melanoma: Current status. Ocul Oncol Pathol (2014) 1:54–62. doi: 10.1159/000367715 27175362 PMC4864524

[50] Chattopadhyay C Kim DW Gombos DS Oba J Qin Y Williams MD . Uveal melanoma: From diagnosis to treatment and the science in between. Cancer (2016) 122:2299–312. doi: 10.1002/cncr.29727 PMC5567680 26991400

[51] Pralhada Rao R Vaidyanathan N Rengasamy M Mammen Oommen A Somaiya N Jagannath MR . Sphingolipid metabolic pathway: an overview of major roles played in human diseases. J Lipids (2013) 2013:178910. doi: 10.1155/2013/178910 23984075 PMC3747619

[52] Niederkorn JY Mellon J Pidherney M Mayhew E Anand R . Effect of anti-ganglioside antibodies on the metastatic spread of intraocular melanomas in a nude mouse model of human uveal melanoma. Curr Eye Res (1993) 12:347–58. doi: 10.3109/02713689308999459 8319494

[53] Wunder CC Welch RC . Femur-bending properties as influenced by gravity: II. ultimate load, moment, and stress for 3-G mice. Aviat Space Environ Med (1977) 48:734–6. 889547

[54] Hannun YA Obeid LM . Sphingolipids and their metabolism in physiology and disease. Nat Rev Mol Cell Biol (2018) 19:175–91. doi: 10.1038/nrm.2017.107 PMC5902181 29165427

[55] Babahosseini H Roberts PC Schmelz EM Agah M . Roles of bioactive sphingolipid metabolites in ovarian cancer cell biomechanics. Annu Int Conf IEEE Eng Med Biol Soc (2012) 2012:2436–9. doi: 10.1109/EMBC.2012.6346456 23366417

[56] Suh DH Kim HS Kim B Song YS . Metabolic orchestration between cancer cells and tumor microenvironment as a co-evolutionary source of chemoresistance in ovarian cancer: a therapeutic implication. Biochem Pharmacol (2014) 92:43–54. doi: 10.1016/j.bcp.2014.08.011 25168677

[57] Park KS Kim MK Lee HY Kim SD Lee SY Kim JM . S1P stimulates chemotactic migration and invasion in OVCAR3 ovarian cancer cells. Biochem Biophys Res Commun (2007) 356:239–44. doi: 10.1016/j.bbrc.2007.02.112 17349972

[58] Jatooratthawichot P Talabnin C Ngiwsara L Rustam YH Svasti J Reid GE . Effect of expression of human glucosylceramidase 2 isoforms on lipid profiles in COS-7 cells. Metabolites (2020) 10:488. doi: 10.3390/metabo10120488 33261081 PMC7761373

[59] Chueakwon P Jatooratthawichot P Talabnin K Ketudat Cairns JR Talabnin C . Inhibition of ceramide glycosylation enhances cisplatin sensitivity in cholangiocarcinoma by limiting the activation of the ERK signaling pathway. Life (Basel) (2022) 12:351. doi: 10.3390/life12030351 35330102 PMC8949529

[60] Zarate YA Hopkin RJ . Fabry's disease. Lancet (2008) 372:1427–35. doi: 10.1016/S0140-6736(08)61589-5 18940466

[61] Walcher L Kistenmacher AK Suo H Kitte R Dluczek S Strauß A . Cancer stem cells-origins and biomarkers: Perspectives for targeted personalized therapies. Front Immunol (2020) 11:1280. doi: 10.3389/fimmu.2020.01280 32849491 PMC7426526

[62] Qian Y Chen X . Senescence regulation by the p53 protein family. Methods Mol Biol (2013) 965:37–61. doi: 10.1007/978-1-62703-239-1_3 23296650 PMC3784259

[63] Lentz KJ Burns RP Loeffler K Feeney-Burns L Berkelhammer J Hook RR Jr . Uveitis caused by cytotoxic immune response to cutaneous malignant melanoma in swine: destruction of uveal melanocytes during tumor regression. Invest Ophthalmol Vis Sci (1983) 24:1063–9. 6874271

[64] Yang W Chen PW Li H Alizadeh H Niederkorn JY . PD-L1: PD-1 interaction contributes to the functional suppression of T-cell responses to human uveal melanoma cells *in vitro* . Invest Ophthalmol Vis Sci (2008) 49:2518–25. doi: 10.1167/iovs.07-1606 PMC2465808 18296654

[65] Goslings WR Blom DJ de Waard-Siebinga I van Beelen E Claas FH Jager MJ . Membrane-bound regulators of complement activation in uveal melanomas. CD46, CD55, and CD59 in uveal melanomas. Invest Ophthalmol Vis Sci (1996) 37:1884–91. 8759358

[66] Owusu BY Galemmo R Janetka J Klampfer L . Hepatocyte growth factor, a key tumor-promoting factor in the tumor microenvironment. Cancers (Basel) (2017) 9:35. doi: 10.3390/cancers9040035 28420162 PMC5406710

[67] Tower H Ruppert M Britt K . The immune microenvironment of breast cancer progression. Cancers (Basel) (2019) 11:1375. doi: 10.3390/cancers11091375 31527531 PMC6769749

[68] Yuan Q Ren J Chen X Dong Y Shang D . Contributions and prognostic performances of m7G RNA regulators in pancreatic adenocarcinoma. Chin Med J (Engl) (2022). doi: 10.1097/CM9.0000000000002179 PMC9746722 36201641

[69] Yuan Q Deng D Pan C Ren J Wei T Wu Z . Integration of transcriptomics, proteomics, and metabolomics data to reveal HER2-associated metabolic heterogeneity in gastric cancer with response to immunotherapy and neoadjuvant chemotherapy. Front Immunol (2022) 13:951137. doi: 10.3389/fimmu.2022.951137 35990657 PMC9389544

[70] Chen X Yuan Q Liu J Xia S Shi X Su Y . Comprehensive characterization of extracellular matrix-related genes in PAAD identified a novel prognostic panel related to clinical outcomes and immune microenvironment: A silico analysis with *in vivo* and vitro validation. Front Immunol (2022) 13:985911. doi: 10.3389/fimmu.2022.985911 36311789 PMC9606578

[71] Chi H Xie X Yan Y Peng G Strohmer DF Lai G . Natural killer cell-related prognosis signature characterizes immune landscape and predicts prognosis of HNSCC. Front Immunol (2022) 13:1018685. doi: 10.3389/fimmu.2022.1018685 36263048 PMC9575041

[72] Wu XZ Shi XY Zhai K Yi FS Wang Z Wang W . Activated naïve b cells promote development of malignant pleural effusion by differential regulation of T(H)1 and T(H)17 response. Am J Physiol Lung Cell Mol Physiol (2018) 315:L443–55. doi: 10.1152/ajplung.00120.2018 29847991

[73] Hao Z Wang S Zheng Z Li J Fu W Han D . Prognostic bone metastasis-associated immune-related genes regulated by transcription factors in mesothelioma. BioMed Res Int (2022) 2022:9940566. doi: 10.1155/2022/9940566 35127947 PMC8813231

[74] Luo T Li Y Nie R Liang C Liu Z Xue Z . Development and validation of metabolism-related gene signature in prognostic prediction of gastric cancer. Comput Struct Biotechnol J (2020) 18:3217–29. doi: 10.1016/j.csbj.2020.09.037 PMC7649605 33209209

[75] Maltby S Khazaie K McNagny KM . Mast cells in tumor growth: angiogenesis, tissue remodelling and immune-modulation. Biochim Biophys Acta (2009) 1796:19–26. doi: 10.1016/j.bbcan.2009.02.001 19233249 PMC2731828

[76] Saakyan SV Zakharova GP Myakoshina EB . Mast cells in the microenvironment of uveal melanoma. Arkh Patol (2019) 81:63–70. doi: 10.17116/patol20198106163 31851194

[77] Bosch JJ Iheagwara UK Reid S Srivastava MK Wolf J Lotem M . Uveal melanoma cell-based vaccines express MHC II molecules that traffic *via* the endocytic and secretory pathways and activate CD8+ cytotoxic, tumor-specific T cells. Cancer Immunol Immunother (2010) 59:103–12. doi: 10.1007/s00262-009-0729-0 PMC2800822 19557412

[78] Ma J Usui Y Takeuchi M Okunuki Y Kezuka T Zhang L . Human uveal melanoma cells inhibit the immunostimulatory function of dendritic cells. Exp Eye Res (2010) 91:491–9. doi: 10.1016/j.exer.2010.06.025 20624388

[79] Wessely A Steeb T Erdmann M Heinzerling L Vera J Schlaak M . The role of immune checkpoint blockade in uveal melanoma. Int J Mol Sci 21 (2020) 21:879. doi: 10.3390/ijms21030879 PMC7037664 32013269

[80] Johnson DB Bao R Ancell KK Daniels AB Wallace D Sosman JA . Response to anti-PD-1 in uveal melanoma without high-volume liver metastasis. J Natl Compr Canc Netw (2019) 17:114–7. doi: 10.6004/jnccn.2018.7070 PMC8063157 30787124

[81] Schank TE Hassel JC . Immunotherapies for the treatment of uveal melanoma-history and future. Cancers (Basel) (2019) 11:1048. doi: 10.3390/cancers11081048 31344957 PMC6721437

[82] Ma D Niederkorn JY . Transforming growth factor-beta down-regulates major histocompatibility complex class I antigen expression and increases the susceptibility of uveal melanoma cells to natural killer cell-mediated cytolysis. Immunology (1995) 86:263–9. PMC1384005 7490128

[83] Ericsson C Seregard S Bartolazzi A Levitskaya E Ferrone S Kiessling R . Association of HLA class I and class II antigen expression and mortality in uveal melanoma. Invest Ophthalmol Vis Sci (2001) 42:2153–6. 11527924

[84] Ho CS Yee AC McPherson R . Complications of surgical and percutaneous nonendoscopic gastrostomy: review of 233 patients. Gastroenterology (1988) 95:1206–10. doi: 10.1016/0016-5085(88)90351-4 3139486

[85] Bosch JJ Thompson JA Srivastava MK Iheagwara UK Murray TG Lotem M . MHC class II-transduced tumor cells originating in the immune-privileged eye prime and boost CD4(+) T lymphocytes that cross-react with primary and metastatic uveal melanoma cells. Cancer Res (2007) 67:4499–506. doi: 10.1158/0008-5472.CAN-06-3770 17483366

