# Corrigendum: Machine learning to construct sphingolipid metabolism genes signature to characterize the immune landscape and prognosis of patients with uveal melanoma

**DOI:** 10.3389/fendo.2024.1421538

**Published:** 2024-05-09

**Authors:** Hao Chi, Gaoge Peng, Jinyan Yang, Jinhao Zhang, Guobin Song, Xixi Xie, Dorothee Franziska Strohmer, Guichuan Lai, Songyun Zhao, Rui Wang, Fang Yang, Gang Tian

**Affiliations:** ^1^ Clinical Medical College, Southwest Medical University, Luzhou, China; ^2^ School of Stomatology, Southwest Medical University, Luzhou, China; ^3^ Department of General, Visceral, and Transplant Surgery, Ludwig-Maximilians-University Munich, Munich, Germany; ^4^ Department of Epidemiology and Health Statistics, School of Public Health, Chongqing Medical University, Chongqing, China; ^5^ Department of Neurosurgery, Wuxi People’s Hospital Affiliated to Nanjing Medical University, Wuxi, China; ^6^ Department of Ophthalmology, Charité – Universitätsmedizin Berlin, Campus Virchow-Klinikum, Berlin, Germany; ^7^ Department of Laboratory Medicine, The Affiliated Hospital of Southwest Medical University, Luzhou, China

**Keywords:** sphingolipid metabolism, UVM, tumor microenvironment, immunotherapy, predictive signature

In the published article, there was an error. There were missing values for the optimal penalty parameter.

A correction has been made to the section Method, subsection Model construction and validation, This sentence previously stated:

“By performing univariate Cox regression analysis, we identified 27 genes associated with survival, followed by Least absolute shrinkage and selection operator (LASSO) regression analysis using ‘glmnet’ in R and using tenfold cross-validation to determine the penalty regularization parameter λ.”

The corrected sentence appears below.

“By performing univariate Cox regression analysis, we identified 27 genes associated with survival, followed by Least absolute shrinkage and selection operator (LASSO) regression analysis using ‘glmnet’ in R, with tenfold cross-validation to determine the optimal penalty parameter lambda.min=9.”

There was another error. The source of the SVM-RFE algorithm was omitted.

A correction has been made to to the section Method, subsection Model construction and validation. This sentence previously stated:

“Using the SVM-RFE algorithm, we obtained 13 valuable variables.”

The corrected sentence appears below.

“We used the SVM-RFE algorithm from the ‘e1071’ R package, with ten-fold cross-validation to obtain 13 valuable variables.”

The authors apologize for these errors and state that this does not change the scientific conclusions of the article in any way. The original article has been updated.

