# Supplementary figures and images for: Machine learning to construct sphingolipid metabolism genes signature to characterize the immune landscape and prognosis of patients with uveal melanoma
Source: Front Endocrinol (Lausanne). 2022 Dec 8;13:1056310. doi: 10.3389/fendo.2022.1056310 (PMC9772281; doi:10.3389/fendo.2022.1056310)

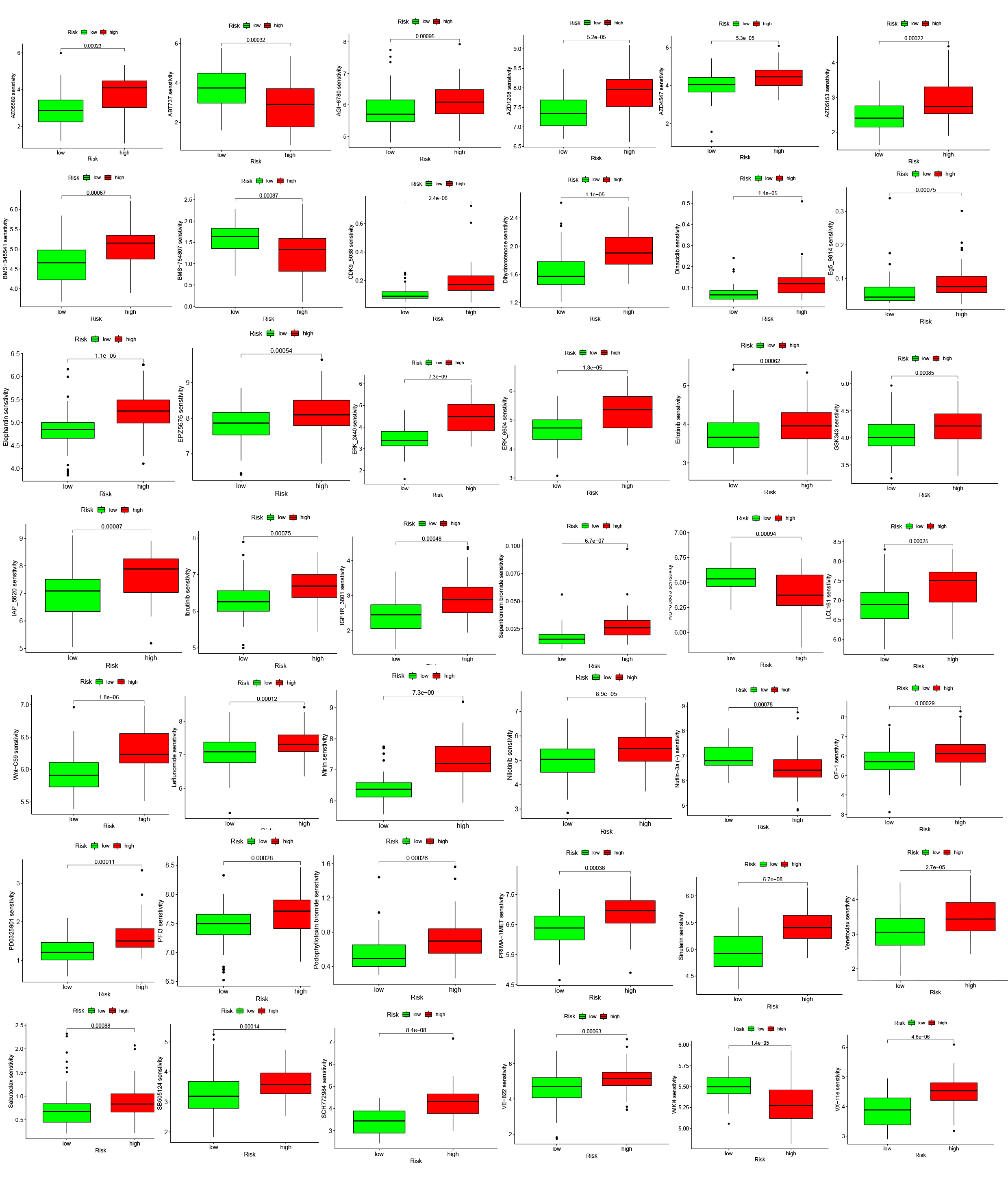

Supplement: Supplementary file 1 [file Image_1.tif]
